# Redistribution of H3K27me3 and acetylated histone H4 upon exposure to azacitidine and decitabine results in de-repression of the AML1/ETO target gene *IL3*

**DOI:** 10.4161/epi.27322

**Published:** 2013-12-02

**Authors:** Francesca Buchi, Erico Masala, Alessia Rossi, Ana Valencia, Elena Spinelli, Alessandro Sanna, Antonella Gozzini, Valeria Santini

**Affiliations:** Hematology; University of Florence; AOU Careggi; Florence, Italy

**Keywords:** H3K27me3, acetylated H4, AML1/ETO, azacitidine, decitabine, acute myeloid leukemia

## Abstract

Human acute myeloid leukemia is characterized by a block in maturation caused by genetic and epigenetic alterations. We studied the effects of low concentrations of the DNA methyltransferase (DNMT) inhibitors 5-azacitidine and decitabine on apoptosis and on chromatin remodeling in an AML1/ETO inducible model of human AML. While both DNMT inhibitors induced apoptosis, only azacitidine did so via caspase activation, possibly through its exclusive non-DNA depending effects. We evaluated histone marks for permissive chromatin, H3K4me3, and acetylated histone H4, and for non-permissive chromatin, H3K9me2, and H3K27me3, at the promoter of the *IL3* gene, which is under the direct control of AML1/ETO and is critical for myeloid maturation. We observed that low concentrations of DNMT inhibitors induced a loss of H3K27me3 and gain of acetylated histone H4 at the *IL3* promoter exclusively in AML1/ETO-positive cells, which was associated with transcriptional reactivation of the *IL3* gene.

## Introduction

Human leukemias are characterized by the coexistence of genetic and epigenetic alterations. The role of histone modifications in the modulation of gene expression in these neoplasms is not yet fully understood. We focused on histone marks associated with permissive and non-permissive chromatin states in a model of acute leukemia. The translocation t(8;21)(q22;q22) is one of the most frequent nonrandom cytogenetic aberrations occurring in acute myeloid leukemia (AML) with granulocytic differentiation and is associated with a relatively favorable prognosis.^1^ This translocation leads to the fusion between the N-terminal portion of *AML1* on chromosome 21 and full-length eight-21 (*ETO*) on chromosome 8, producing the chimeric gene *AML1/ETO,* which leads to the silencing of myeloid maturation genes and is responsible, together with additional mutagenic events, for the leukemic phenotype.^2^ The transcription factor AML1, which binds proteins such as the lysine acetyltransferases p300 and CREB-binding protein (CBP), plays an important role in hematopoiesis as a regulator of the expression of hematopoietic-specific genes, including interleukin 3 (*IL3*),^3^ granulocyte-macrophage colony stimulating factor (*GM-CSF, CSF2*),^4^ myeloperoxidase (*MPO*), neutrophil elastase (*ELANE*),^5^ and macrophage colony-stimulating factor (*M-CSF, CSF1*)^6^ while interacting with transcription factors such as PU.1 and CCAAT/enhancer binding protein, α (CEBPA).^7^ CBP and p300 cofactors promote acetylation of histones, a feature associated with active transcription.^8^ The fusion protein AML1/ETO recruits histone deacetylases (HDACs)^9^ and DNA methyltransferase-1 (DNMT1)^10^ to AML1 DNA binding sites and acts as a potent negative regulator of transcription of the genes normally controlled by AML1.^11^^-^^13^ The epigenetic mechanisms involved in AML1/ETO leukemogenesis prompted us to investigate a possible selective role of epigenetic agents in AML carrying this chimeric protein. We previously demonstrated that low concentrations of the HDAC inhibitor givinostat (ITF2357) have selective anti-leukemic activity on AML1/ETO-positive cells, inducing apoptosis and altering the intracellular localization of p300 and DNMT1.^14^

Methylation and acetylation of histones H3 and H4 contribute to determining transcriptional accessibility. Trimethylation of lysine 4 of histone H3 (H3K4me3) and acetylation of histone H4 can be markers of euchromatin associated with active gene expression, and dimethylation of lysine 9 (H3K9me2) and trimethylation of lysine 27 (H3K27me3) can be markers of heterochromatin associated with gene silencing.^15^ We evaluated the histone marks H3K4me3, acetylation of histone H4, H3K9me2, and H3K27me3 in leukemic cells expressing and not expressing AML1/ETO with and without exposure to the DNMT inhibitors, 5-azacitidine (AZA) and decitabine (DAC). At the dose currently used in clinics, these agents have been demonstrated to induce general DNA hypomethylation, and because of their clinical efficacy^16^^,^^17^ are at present the standard of care in many countries for the treatment of myelodysplastic syndromes (MDS) and elderly AML, although the response to treatment shown by patients varies widely. We demonstrate here that in vitro exposure to very low concentrations of DNMT inhibitors can revert AML1/ETO-mediated gene silencing with high specificity, redistributing histone marks, in particular H3K27me3, and induce growth inhibitory and pro-apoptotic effects that are greater in cells that express the oncogenic protein than in those that do not.

## Results

### Effects of DNMT inhibitors on cell viability and apoptosis

U937-A/E-9/14/18 cells were incubated with 5 μM of ponasterone A for 48 h to induce AML1/ETO expression and then with AZA or DAC at different concentrations for 24 h. AML1/ETO expression was confirmed by western blot analysis. We observed that neither AZA nor DAC induced changes in AML1/ETO protein expression (Fig. 1A and B).

**Figure:**
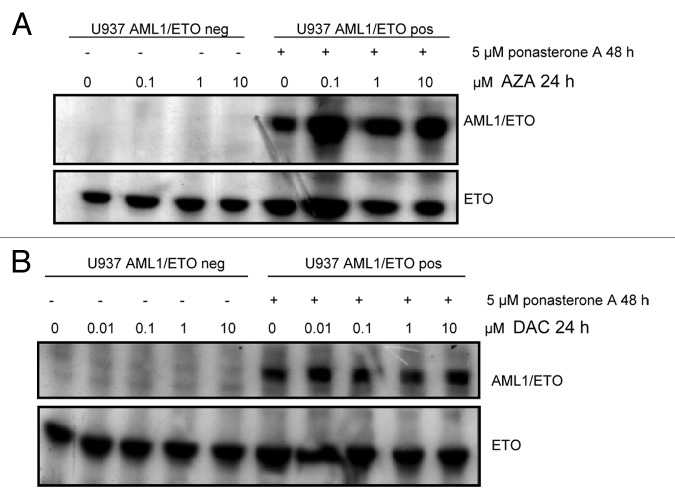
**Figure 1.** Conditional expression of AML1/ETO protein. Evaluation of the expression of AML1/ETO in U937-A/E-9/14/18 cells in the absence or presence of 5 μM ponasterone A for 48 h and after exposure to AZA (**A**) or DAC (**B**) at the indicated doses. Cells were lysed, and western blot analysis was performed with anti-ETO antibody. neg, negative; pos, positive.

We then evaluated the concentration-related effects of AZA and DAC exposure on cell viability and apoptosis in U937 cells with or without AML1/ETO expression induced. AZA 0.1 µM significantly reduced the viability (80% viable cells relative to untreated cells) of both AML1/ETO-negative and -positive cells (Fig. S1A). DAC 0.01 μM produced an equivalent effect in AML1/ETO-positive cells, but in AML1/ETO-negative cells, a similar effect was observed only with DAC 10 μM (Fig. S1B).

In AML1/ETO-positive cells AZA and DAC were more active in inducing apoptosis than in AML1/ETO negative cells (Fig. 2A and B). In fact, in AML1/ETO-positive cells a significant increase in apoptosis was observed after exposure to AZA 0.1 µM and DAC 0.01 μM (treated vs control, *P* < 0.01), whereas in AML1/ETO-negative cells, a significant effect was achieved with AZA 1 μM and DAC 0.1 μM (treated vs control, *P* < 0.01) (Fig. 2A and B). We then observed that AZA, but not DAC, induced caspase activation (Fig. 2C and D). In particular, cleaved caspase 9 appeared after AZA 1 µM treatment in AML1/ETO-positive cells and after AZA 10 μM treatment in AML1/ETO-negative cells. Caspase 8 (18 KDa) was cleaved after AZA 0.1 µM treatment only in AML1/ETO-positive cells, and the 2 cleaved fragments (17–19 KDa) of caspase 3 were observed after AZA 10 µM treatment in AML1/ETO-negative cells and after AZA 1 µM in AML1/ETO-positive cells (Fig. 2C).

**Figure:**
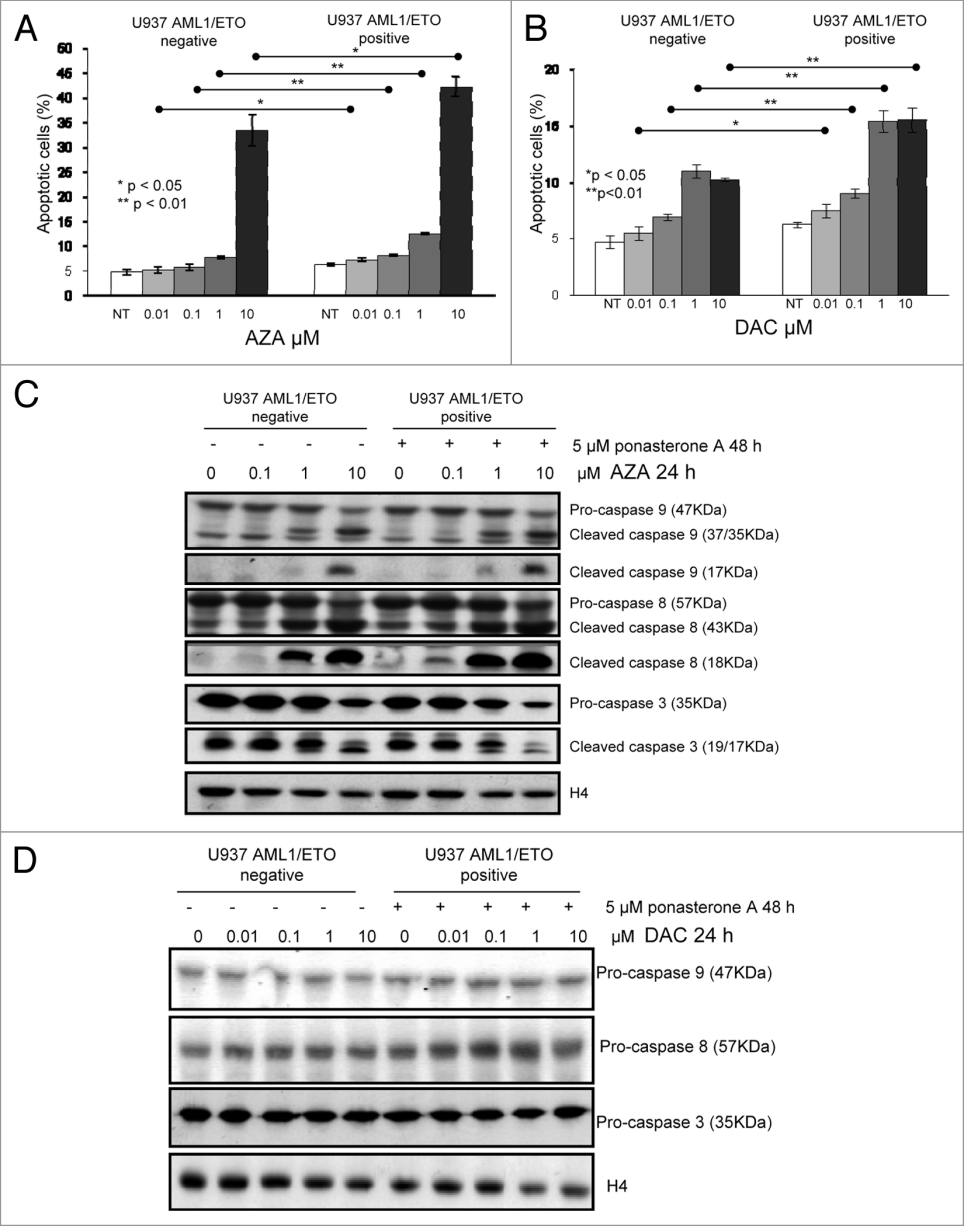
**Figure 2.** Effects of DNMT inhibitors AZA and DAC on apoptosis. U937-A/E-9/14/18 cells, in the absence or the presence of 5 μM ponasterone A for 48 h, were exposed to AZA or DAC at the indicated doses for 24 h. (**A and B**) Apoptosis was measured by the Annexin-V test, and the percentages of apoptotic cells are reported. Data represent the average ± standard deviation of three independent experiments. Significance between AML1/ETO positive and negative cells has been calculated by the Mann-Whitney test**;** (* *P* < 0.05, ** *P* < 0.01); (**C and D**). Caspase cleavage was also analyzed. Cells were lysed, and western blot analysis was performed with the indicated antibodies. Equalization of protein loading was verified on the same membrane by stripping and incubating with anti-H4 antibody. NT, not treated.

### Effects of DNMT inhibitors on histone marks at the promoter of *IL3*

We evaluated the modulating activity of AZA and DAC (used at the minimal effective concentrations established with the above-mentioned demonstration of apoptosis) on the levels of acetylated histone H4 and H3K4me3 (as markers of euchromatin), H3K9me2, and H3K27me3 (as markers of heterochromatin) in whole cell lysates of AML1/ETO-positive and negative cells (Fig. S2). Exposure to either DNMT inhibitor did not lead to significant modulation of any histone marks. We thus reckoned that the best approach was to evaluate regional specific changes of histone marks, i.e., at *IL3* promoter, which is under the transcriptional control of AML1 and AML1/ETO.

Therefore we examined chromatin properties specifically at the *IL3* promoter site, using a chromatin immunoprecipitation (ChIP) assay. *IL3* promoter is characterized by the presence of two binding sites for AML1 and AML1/ETO placed at –192 bp (TGTGGT) and –105 bp (TGTGGG) from the start site of transcription. Our primers were designed to amplify a region including both binding sites (Fig. 3A). Antibodies against acetylated histone H4, H3K4me3, H3K9me2, and H3K27me3 were used to co-immunoprecipitate the *IL3* promoter in inducible AML1/ETO cells, antibody against acetylated histone H4 to co-immunoprecipitate *IL3* promoter in HL60 cells and in AML1/ETO positive Kasumi-1 cells, with and without AZA or DAC exposure (Fig. 3B–D). In U937cells not exposed to DNMT inhibitors, only H3K27me3 and H3K9me2 were able to co-immunoprecipitate the *IL3* promoter but not acetylated histone H4 or H3K4me3 (Fig. 3B, lanes 1 and 4), indicating non-permissive chromatin in this region. In cells exposed to low concentrations of AZA or DAC, we observed co-immunoprecipitation of the *IL3* promoter with acetylated histone H4 only in U937 AML1/ETO-positive cells. Moreover, in the same cells, both AZA and DAC exposure led to the reversal of *IL3* promoter co-immunoprecipitation with H3K27me3 (Fig. 3B).

**Figure:**
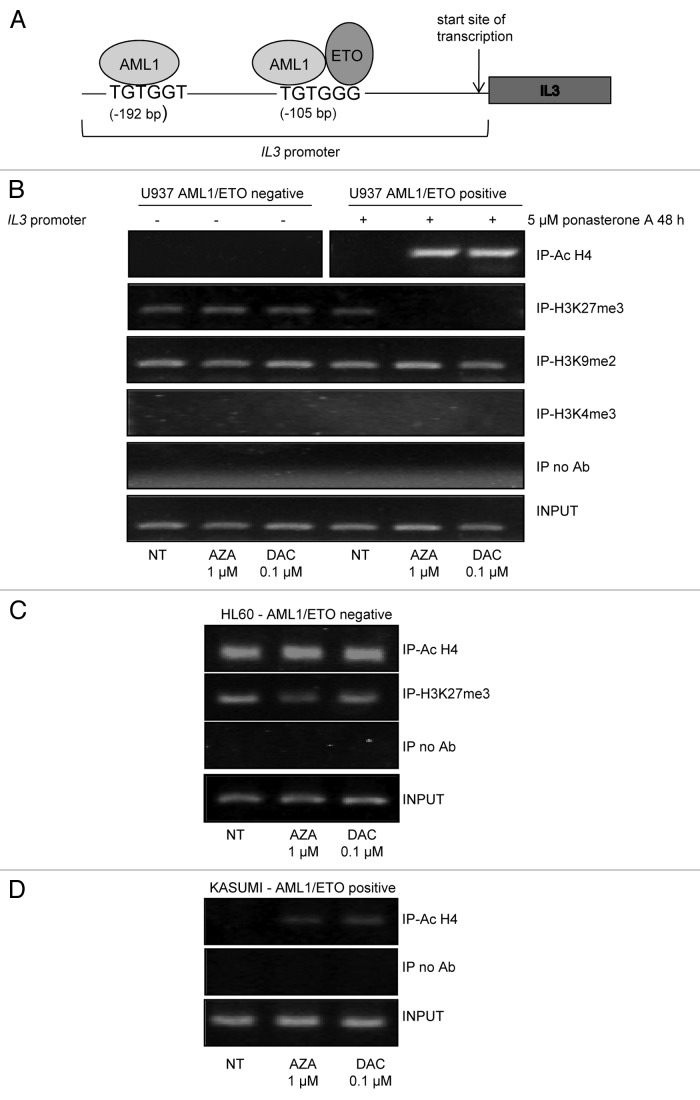
**Figure 3.** Effects of DNMT inhibitors AZA and DAC on histone marks at the *IL3* promoter**.** (**A**) Schematic representation of *IL3* promoter, characterized by the presence of two binding sites for AML1 and AML1/ETO proteins placed at -192 bp (TGTGGT) and -105 bp (TGTGGG) from the start site of transcription. Our primers were designed to amplify a region including both binding sites. (**B–D**) U937-A/E-9/14/18 cells, in the absence or the presence of 5 μM ponasterone A for 48 h (**B**), HL60 (**C**) and Kasumi-1 (**D**) were exposed to AZA or DAC for 24 h at the indicated doses. ChIP assay with indicated antibodies was performed, and PCR to test *IL3* expression was performed. Ab, antibody; Ac, acetylated; IP, immunoprecipitated; NT, not treated; INPUT, positive control.

Consistent with the results observed for cell growth and apoptosis, in AML1/ETO-negative cells, treatment with low concentrations of DNMT inhibitors did not modify H3K27me3 association with the *IL3* promoter and did not induce markers of permissive chromatin (Fig. 3B).

The association of the other two histone modifications, H3K4me3 or H3K9me2, with the *IL3* promoter was not affected by treatment with AZA or DAC in either AML1/ETO-negative or -positive cells, possibly indicating their relative lack of significance for transcription in this setting.

To confirm these observations, we employed two leukemic cells lines: non-expressing AML1/ETO (HL60) and expressing AML1/ETO (Kasumi-1). In HL60 myeloid cells AZA and DAC did not modify co-immunoprecipitation pattern of *IL3* with acetylated histone H4 and H3K27me3 (Fig. 3C), while in Kasumi-1 cells, treatment with both drugs was able to induce histone H4 co-immunoprecipitation, possibly indicating reversion of chromatin accessibility on this site (Fig. 3D).

### Effects of DNMT inhibitors on *IL3* expression

To assess the validity of our model, we evaluated *IL3* mRNA expression in AML1/ETO-expressing and -non-expressing cells after treatment with ponasterone A 5 μM for 48 h. We confirmed that AML1/ETO inhibited *IL3* mRNA expression (Fig. 4A). Treatment with AZA 1 μM or DAC 0.1 μM for 24 h increased *IL3* mRNA expression in both AML1/ETO-negative and -positive cells, but this effect was significant only in AML1/ETO-positive cells (Fig. 4B).

**Figure:**
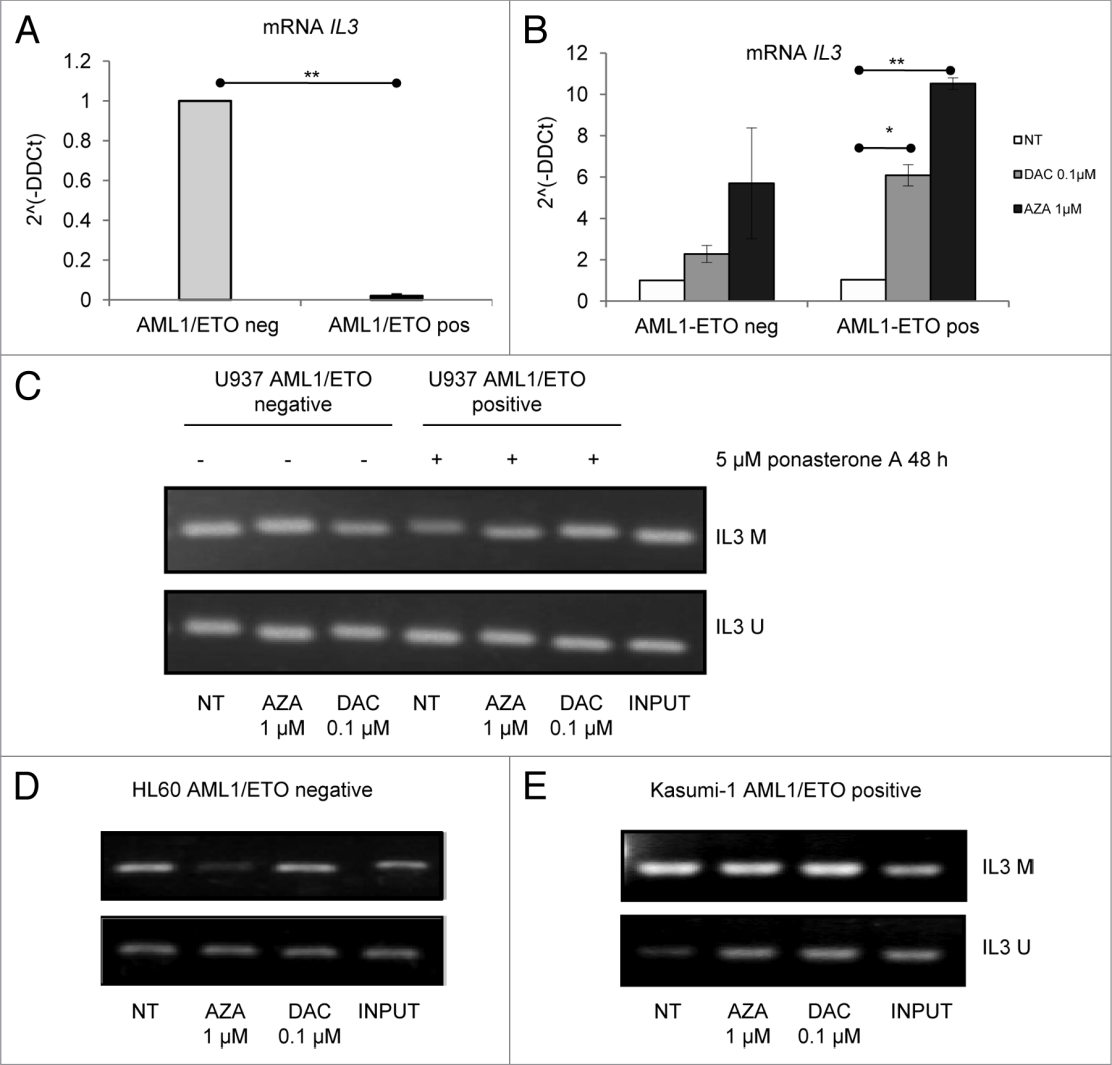
**Figure 4.** Effects of DNMT inhibitors AZA and DAC on mRNA expression and promoter methylation of *IL3*. U937-A/E-9/14/18 in the absence or the presence of 5 μM ponasterone A for 48 h, HL60, and Kasumi-1 cells were exposed to AZA and DAC for 24 h at the indicated doses. (**A and B**) RNA was extracted and cDNA obtained by reverse transcription; the expression of *IL3* mRNA was evaluated by quantitative real-time PCR and calculated by a comparative threshold cycle method, using the Equation 2^(-ΔΔCt). (**A**) Relative expression of *IL3* mRNA in AML1/ETO-positive/-negative cells in the absence or presence of 5 μM ponasterone A for 48 h. (**B**) Relative expression of *IL3* mRNA in AML1/ETO-positive/-negative cells after exposure to AZA and DAC and with not treated (NT) cells. (**C–E**) DNA was extracted and modified by sodium bisulfite treatment, the presence of methylated (M) and unmethylated (U) *IL3* promoter was evaluated by methylation specific PCR in U937-A/E-9/14/18 (**C**), HL60 (**D**), and Kasumi-1cells (**E**). INPUT, universal methylated (upper) and unmethylated (lower) DNA; neg, negative; pos, positive.

### Effects of DNMT inhibitors on *IL3* promoter methylation

To investigate the mechanisms of re-expression of the *IL3* gene, we evaluated *IL3* promoter methylation by methylation-specific polymerase chain reaction (PCR) in AML1/ETO-negative and -positive U937 cells (Fig. 4C) and, as further validation, in AML1/ETO-negative HL60 cells (Fig. 4D) and AML1/ETO positive leukemic Kasumi-1 cells (Fig. 4E). The *IL3* promoter was not observed to be hypermethylated at baseline, and neither AZA nor DAC treatments affected methylation levels of the *IL3* promoter in any cellular model analyzed.

## Discussion

Alterations of chromatin organization are a common mechanism of oncogenesis,^18^ and so-called “epigenetic” drugs have shown great efficacy in the treatment of hematopoietic neoplasms.^16^^,^^17^^,^^19^ We have previously demonstrated a selective anti-leukemic activity of the HDAC inhibitor givinostat (ITF2357) on AML1/ETO-positive cells.^14^ This cellular and molecular model is particularly intriguing because it may constitute a selective target for epigenetic therapy. In fact, AML1/ETO forms a multiprotein complex with HDACs^9^ and DNMT1,^10^ resulting in the stable silencing of AML1-controlled genes, such as *IL3*. Thus we hypothesized that the DNMT inhibitors AZA and DAC could have a targeted effect on AML1/ETO-positive leukemic cells, and be active at lower doses. We in fact observed that loss of H3K27me3 and gain of acetylated H4 at the *IL3* promoter region in AML1/ETO-positive cells exposed to the lowest biologically effective concentrations of DNMT inhibitors was associated with transcriptional activation, consistent with a loss of AML1/ETO-mediated repression. Such histone mark modulation was exclusively taking place at the specific DNA region controlled by AML1 and was not evident when whole histone evaluation was performed.

We first confirmed that DNMT inhibitors were able to induce apoptosis of AML1/ETO-positive cells at minimal concentrations. Moreover, at these concentrations, AZA and DAC did not lead to diffuse DNA hypomethylation, confirming what has been previously reported,^20^^,^^21^ but, while methylation of the specific promoter region analyzed here did not change, chromatin reorganization in this same region was observed after exposure to either agent.

AZA and DAC mechanisms of action have not yet been fully elucidated or differentiated. From our results, it is clear that the two agents show an in vitro difference in the ability to induce apoptosis as well as in the efficiency of gene derepression. In fact, AZA promoted significantly higher expression of *IL3* than DAC. Our observations could be possibly related to the RNA incorporation of AZA and its inhibitory effect on protein synthesis.^22^^,^^23^ On the other hand, redistribution of histone marks was the same with AZA or DAC.

It has been shown that DNA methylation affects histone modifications and hence chromatin structure;^24^ however, the “hierarchy” of such molecular events remain incompletely understood. To date, the effects of DNMT inhibitors on histone modification in human leukemic models have not been studied. We studied histone marks associated with the *IL3* promoter region because this gene is under the direct repressive control of AML1/ETO, and it is critical for myeloid maturation.^3^ As alluded to above, we observed that both drugs induced a change in chromatin properties only in AML1/ETO-positive cells. Indeed, AZA and DAC treatment led to the acetylation of histone H4 (a general marker of euchromatin)^15^ only in the AML1/ETO positive Kasumi-1 cells, as well as in U937 cells expressing AML1/ETO, and in the latter to the loss of the histone mark H3K27me3, both at the *IL3* promoter. Histone H4 co-immunoprecipitated with *IL-3* promoter was not acetylated upon AZA and DAC treatment of U937 and HL 60 cells, not expressing AML1/ETO.

The lack of co-immunoprecipitation of *IL3* promoter and H3K4me3 that we observed in AML1/ETO positive cells after AZA and DAC exposure may be consistent with the complexity of histone mark association with AML1 already described by other Authors.^25^

The reduction of H3K27me3 by itself seemingly could induce re-expression of AML1/ETO-controlled genes. Because of the loss of H3K27me3 (and the gain of acetylated H4) at the *IL3* promoter, we evaluated the expression of the *IL3* gene. We observed that in U937 AML1/ETO-positive cells the relative expression of *IL3* mRNA increased significantly after treatment. However, in AML1/ETO-negative cells, neither AZA nor DAC treatment led to a significant increase in its expression.

The polycomb repressive complex 2 (PRC2)/EZH2, which adds the repressive histone mark H3K27me3, is able to recruit DNMTs.^26^^,^^27^ However, the majority of H3K27me3-marked genes lack proximal DNA methylation, indicating that PRC2/EZH2 recruitment of DNMTs may not be sufficient to promote DNA methylation.^24^^,^^27^ In fact, studies mapping epigenetic modifications in the genome in normal and cancerous tissues have shown that patterns of DNA methylation and H3K27me3 marks are mutually exclusive.^28^^-^^30^

As expected based on these previous observations, at baseline the *IL3* promoter was not hypermethylated in our cell model, and its methylation status did not change after AZA and DAC exposure. Therefore, we demonstrate that in our AML1/ETO model, the H3K27me3 mark, although essential to *IL3* gene silencing, did not affect and was not accompanied by regional DNA hypermethylation, as far as it is measurable by MSP. Our previous findings^14^ are consistent with these data, while more quantitative assays may indeed be sensitive enough to show some regional hypermethylation.^31^

We conclude that modification of the patterns of transcriptional repression may be solely caused by the redistribution of the H3K27me3 mark and the repressive activity of the AML1/ETO complex. Awareness of H3K27me3 as a mark of gene repression in the AML1/ETO model is especially important for the classification of human leukemias using chromatin and histone profiles and may open new horizons for epigenetic therapy. At the same time, further exploring the modulating effects exerted by AZA and DAC at concentrations with not maximal hypomethylating efficiency may further clarify their mode of action and selectivity.

## Materials and Methods

### Cells and culture conditions

AML1/ETO-inducible human myeloid leukemia U937-A/E-9/14/18, AML1/ETO-positive Kasumi-1, and AML1/ETO negative HL60 cell lines were cultured at 3 × 10^5^ cells/mL in RPMI 1640 supplemented with 10% fetal bovine serum (Euroclone), 4 mM glutamine, 50 units/mL penicillin, and 50 µg/mL streptomycin at 37 °C in a humidified atmosphere containing 5% carbon dioxide. The U937 cell line was established as previously described.^32^ Individual U937 cell clones stably cotransfected with an ecdysone-inducible AML1/ETO expression system (kindly provided by M. Lübbert, Germany), were maintained in the presence of 600 μg/ml G418 (Life Technologies) and 150 μg/ml Zeocin (Life Technologies). AML1/ETO expression was induced by adding 5 μM ponasterone A (Sigma Aldrich) daily for 48 h directly to the cell cultures, and western blot was performed to confirm AML1/ETO expression.

All experiments were performed by incubating cells in the presence or absence of different concentrations (as indicated) of 5-Aza-2′-deoxycytidine (Sigma Aldrich) and 5-Azacytidine (Sigma Aldrich) for 24 h.

### Cell viability and apoptosis

Cell viability was evaluated and quantified by cell counting using the trypan blue exclusion method. Apoptosis was evaluated using the Annexin-V-FLUOS Staining Kit (Roche Applied Science), according to the manufacturer’s instructions, and flow cytometry was performed using a FACSscan (Becton and Dickinson). All experiments were performed in triplicate, and standard deviation was calculated.

### Cell lysis and western blotting

Cells lysis and western blotting were performed as previously described.^22^ Membranes were incubated in phosphate-buffered saline (PBS) containing 0.1% Tween 20 and 1% bovine serum albumin (BSA; T-PBS/1% BSA) with a 1:1000 dilution of primary antibody (anti-caspase 9, anti-caspase 8, anti-caspase 3, Cell Signaling; anti-ETO, Santa Cruz Biotechnology; anti-trimethyl-Histone H3 [Lys27], (Upstate, Lot:0701050758, CA), anti-trimethyl-Histone H3 [Lys4] (Millipore, Lot NG1643014,CA), anti-dimethyl-Histone H3 [Lys9] (Upstate, Lot:0608038250, CA), anti-acetyl-Histone H4 (Millipore, Lot: DAM1661056, CA) for 6 to 18 h at 4 °C. Horseradish peroxidase-conjugated secondary antibodies used were anti-rabbit immunoglobulin (Ig) G (1:5000 in T-PBS/2% BSA, Santa Cruz, Lot: F1506, CA), anti-mouse IgG (1:5000 in T-PBS/5% BSA, Santa Cruz Biotechnology, Lot: 323209, CA), or antigoat IgG (1:10000 in T-PBS/5% BSA; Santa Cruz Biotechnology, CA). Antibody-coated protein bands were visualized by ECL chemiluminescence detection (Euroclone). To check equal loading of sample, membranes were incubated in stripping buffer (62.5 mM TRIS-HCl pH 6.7, 2% sodium dodecyl sulfate [SDS], 100 mM 2-mercaptoethanol; 30 min, 50 °C), and then the same membranes were incubated in a 1:1000 dilution of anti-H4 (Millipore, Lot: DAM1661056, CA) and anti tubulin A (Cell Signaling) (in T-PBS/5% BSA (16–18 h at 4 °C).

### RNA extraction, reverse transcription PCR, and quantitative real-time PCR

Total RNA was extracted from cells by TRIzol^®^ reagent (Life Technologies) according to the manufacturer’s instructions. RNA concentration was determined, and cDNA (cDNA) was synthesized from 1 μg of total RNA using the Improm-II^TM^ Reverse Transcription System (Promega) according to the manufacturer’s instructions.

*IL3* mRNA was quantified by quantitative real-time PCR with the ABI Prism 7300 Sequence Detection System (Applied Biosystems) using Power SYBR^®^ Green PCR Master Mix (Applied Biosystems). The housekeeping glyceraldehyde-3-phosphate dehydrogenase (*GAPDH*) gene was used as an internal reference. The primer sequences were as follows:

*IL3-*forward 5′-CCAAACCTGG AGGCATTCA-3′; *IL3-*reverse 5′-TCAATTGCTG ATGCGTTCTG TA-3′; *GAPDH*-forward 5′-AACAGCCTCA AGATCATCAG CAA-3′; *GAPDH*-reverse 5′-CAGTCTGGGT GGCAGTGAT-3′. Primers were used at a final concentration of 200 nM. PCR was performed using following parameters: 95 °C for 10 min, then 40 cycles of an annealing-extension step at 60 °C for 1 min, and denaturation at 95 °C for 15 s. To exclude contamination of nonspecific PCR products, melting curve analysis was always applied to final PCR products after the cycling protocol. The relative expression of *IL3* was calculated using a comparative threshold cycle method, using the Equation 2^(-ΔΔCt). All experiments were performed in triplicate, and the standard deviation was calculated.

### ChIP assay

The ChIP assay was performed as follows. Cells (1 × 10^6^) were treated with 1% formaldehyde for 10 min at 37 °C, centrifuged at 4 °C, and washed using ice-cold PBS containing protease inhibitors (1 mM phenylmethylsulfonyl fluoride, 1 µg/ml aprotinin and 1 µg/ml pepstatin A; Sigma Aldrich). Cell pellets were solubilized in lysis buffer (50 mM TRIS-HCl pH 8.1, 10 mM ethylenediamine-tetra-acetic acid [EDTA], 1% SDS) with protease inhibitors for 10 min on ice and then sonicated to generate DNA fragments of 500 base pairs on average. After centrifugation, the supernatant was diluted with ChIP dilution buffer (16.7 mM TRIS-HCl pH 8.1, 167 mM NaCl, 1.2 mM EDTA, 0.01% SDS, 1.1% Triton X-100). A total of 4% of this sample was used as an indicator of chromatin content in each sample. Antibody (2 µg/sample; anti-trimethyl-Histone H3 (Lys27) (Upstate, Lot:0701050758, CA), anti-trimethyl-Histone H3 (Lys4) (Millipore, Lot: DAM1661056, CA), anti-dimethyl-Histone H3 (Lys9) (Upstate, Lot: 0608038250, CA), anti-acetyl-Histone H4 (Millipore, Lot: DAM1661056, CA), was incubated in a suspension of PBS + 0.02% Tween and 4 μg/μl of Dynabeads^®^ Protein G (Invitrogen) for 30 min to 1 h at room temperature in agitation. Following centrifugation, beads + antibody were resuspended in PBS + BSA 100 mg/mL, added to samples, and incubated overnight at 4 °C in agitation. Each sample was subjected to the same procedure in parallel with beads without antibody (negative control). Antibody-protein-DNA complexes were collected by centrifugation, and after extensive washing, complexes were eluted with 500 µL elution buffer (0.1 M sodium bicarbonate, 1% SDS). Following addition of 5 M sodium chloride, all samples, including input, were incubated at 65 °C for 4 h to revert cross-linking. After treatment with 20 mg/mL RNase and digestion with 40 mM proteinase-K, DNA was purified with system based on silica-membrane spin columns, according to the manufacturer’s recommendations (Wizard^®^ SV Gel and PCR Clean-up System; Promega). DNA obtained by immunoprecipitated chromatin was amplified by PCR performed on a DOPPIO Cycler (VWR) using GoTaq^®^ Hot Start Polymerase (Promega) with the following parameters: 94 °C for 2 min; 45 cycles at 94 °C for 40 s, 57 °C for 40 s, and 72 °C for 40 s; final extension at 72 °C for 5 min. Primers for the *IL3* promoter were: forward 5′-AGTCATGGAT GAATAATTACG-3′; reverse 5′-TCCCGCCTTA TATGTGC-3′. PCR products were separated by electrophoresis on a 2% agarose gel and visualized using EuroSafe Nucleic Acid Stain (Euroclone).

### Methylation-specific PCR

Genomic DNA was extracted from cells and purified using PureLink Genomic DNA (Invitrogen) according to the manufacturer's instructions. Extracted DNA was modified by sodium bisulfite treatment (MethylCode Bisulfite Conversion Kit; Invitrogen) according to the manufacturer's instructions. Methylation-specific PCR was performed on a DOPPIO Cycler using GoTaq^®^ Hot Start Polymerase and primers for the methylated (M) and unmethylated (U) promoter sequences were:

*IL3 M*-forward 5′- ATAAGATAGA GTGTTTTTTG TCGA-3′,

*IL3 M*-reverse 5′-AATCATAAAA ACTTAAAATC CGAA-3′,

*IL3 U*-forward 5′-AATAAGATAG AGTGTTTTTT GTTGA-3′,

*IL3 U*-reverse *5*’-TCTAAATCAT AAAAACTTAA AATCCAAA-3′;

CpGenome Universal Methylated DNA and CpGenome Universal Unmethylated DNA (Millipore) were used as positive controls. PCR was performed using the following parameters: 94 °C for 5 min; 40 cycles at 95 °C for 30 s, 57 °C for 30 s, 72 °C for 1 min; final extension at 72 °C for 5 min. PCR products were separated by electrophoresis on a 2% agarose gel and visualized with EuroSafe Nucleic Acid Stain (Euroclone).

### Statistical analysis

For cell viability, apoptosis assay, and quantitative real-time PCR, the Mann-Whitney test was employed to determine the significance of differences between various experimental conditions.

## Supplementary Material

Additional material

Additional material

## Abbreviations:

AML: acute myeloid leukemia
CBP: CREB-binding protein
*IL3*: interleukin 3
*GM-CSF*: granulocyte macrophage colony stimulating factor
*MPO*: myeloperoxidase
*M-CSF*: macrophage colony stimulating factor
CEBPα: CCAAT/enhancer binding protein α
HDAC: histone deacetylase
DNMT: DNA methyltransferase
H3K4me: trimethylation of lysine 4 of histone H3
H3K9me: dimethylation of lysine 9 of histone H3
H3K27me: trimethylation of lysine 27 of histone H3
AZA: azacitidine
DAC: decitabine
MDS: myelodysplastic syndromes
ChIP: chromatin immunoprecipitation
PCR: polymerase chain reaction
PRC2: polyrepressive complex 2
PBS: phosphate-buffered saline
BSA: bovine serum albumin
Ig: immunoglobulin
SDS: sodium dodecyl-sulfate
EDTA: ethylenediamine-tetra-acetic acid
cDNA: complementary DNA
M: methylated
U: unmethylated

## Notes

10.4161/epi.27322
