# Supplementary material for: Redistribution of H3K27me3 and acetylated histone H4 upon exposure to azacitidine and decitabine results in de-repression of the AML1/ETO target gene IL3
Source: Epigenetics. 2013 Dec 2;9(3):387–95. doi: 10.4161/epi.27322 (PMC4053457; doi:10.4161/epi.27322)
Supplement: Additional material [file epi-9-387-s01.pdf]

Landes Bioscience www.landesbioscience.com

Supplemental Material to:

Francesca Buchi, Erico Masala, Alessia Rossi, Ana Valencia, Elena Spinelli, Alessandro Sanna, Antonella Gozzini, and Valeria Santini

Redistribution of H3K27me3 and acetylated histone H4 upon exposure to azacitidine and decitabine results in de-repression of the AML1/ETO target gene IL3

Epigenetics 2013; 9(3)
http://dx.doi.org/10.4161/epi.27322

http://www.landesbioscience.com/journals/epigenetics/article/27322/

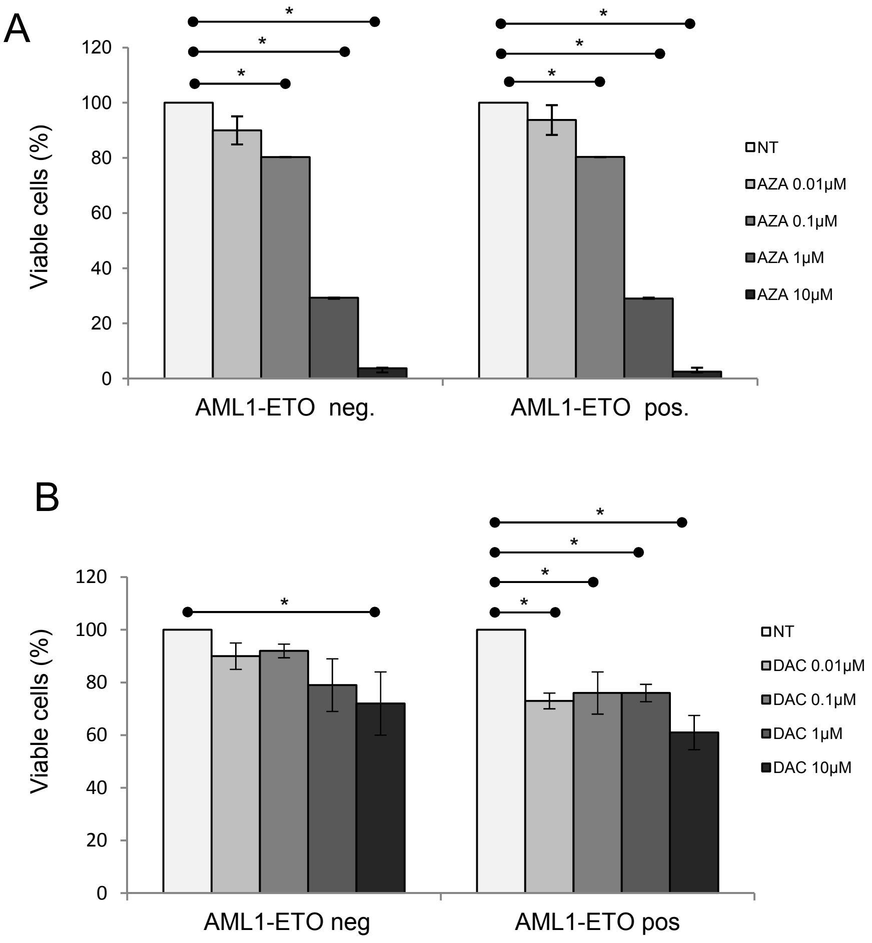

Figure S1. Effects of DNA methyltransferase inhibitors azacitidine and decitabine on cell viability.

U937-A/E-9/14/18 cells, in the absence or the presence of 5 µM ponasterone A for 48 h, were exposed to azacitidine (A) or decitabine (B) at the indicated doses for 24 h. Viable cells were counted by trypan blue exclusion. Data represent the average ± standard deviation of three independent experiments. Significance of differences has been calculated with respect to untreated cells by Mann-Whitney test (* $p < 0.01$).

AML1/ETO: acute myeloid leukemia 1/eight-twenty-one; NT: not treated; AZA: azacitidine; DAC: decitabine.

Figure 1S

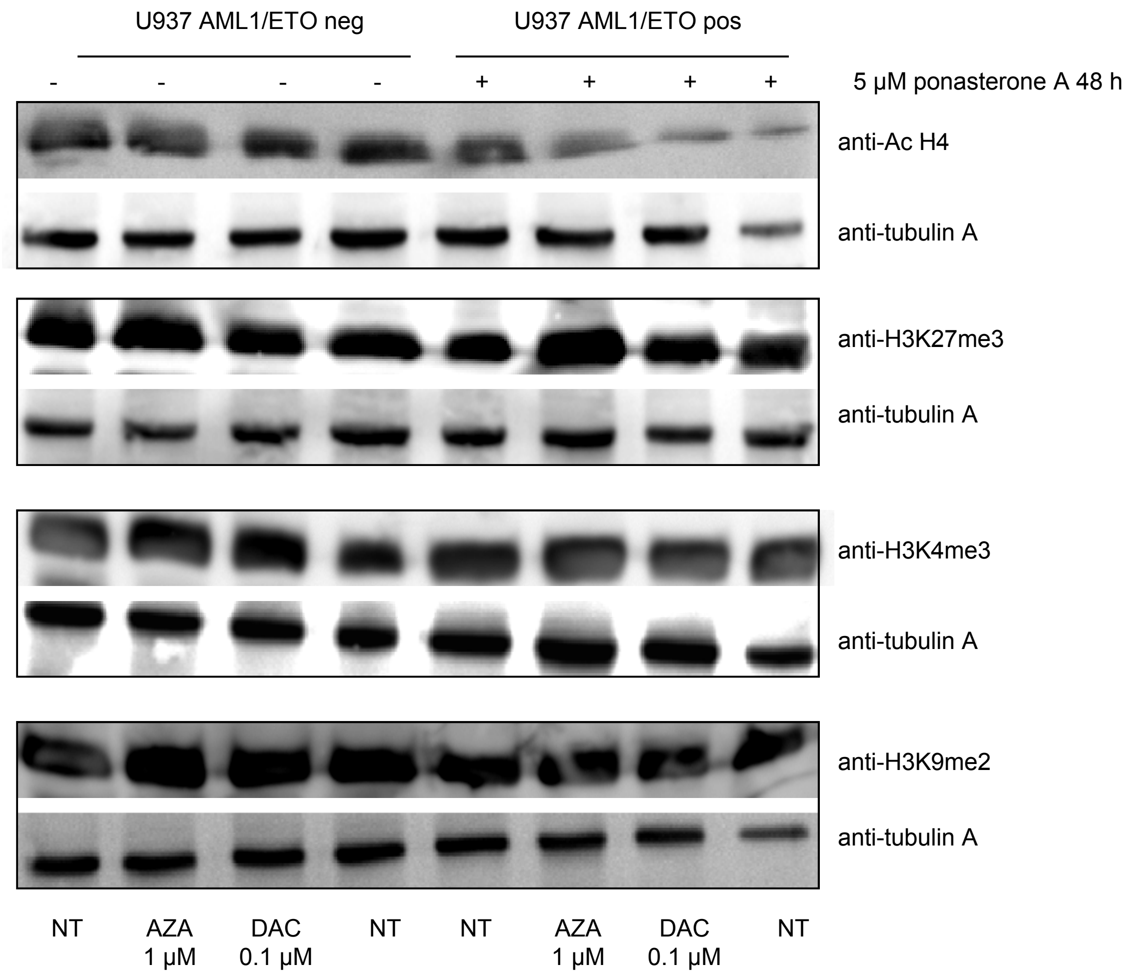

Figure 2S. Effect of DNA methyltransferase inhibitors azacitidine and decitabine on histone modifications.

U937 cells, in the absence or the presence of 5 μM ponasterone A for 48 h, exposed to azacitidine or decitabine at indicated doses for 24 h were lysed and western blot analysis was performed with indicated antibodies. Deacetylation of histone H4 in AML1/ETO expressing cells treated with AZA and DAC parallels their increased sensitivity to the apoptotic/cytotoxic effects of very low doses of both agents.

Equalization of protein loading was verified on the same membrane by stripping and incubating with anti-tubulin A antibody.

Ac: acetylated; AML1/ETO: acute myeloid leukemia 1/eight-twenty-one; H3K4me3: trimethylated lysine 4 on histone H3; H3K9me2: dimethylated lysine 9 on histone H3; H3K27me3: trimethylated lysine 27 on histone H3; NT: not treated; AZA: azacitidine; DAC: decitabine.

Figure 2S
